# Conservatively Managed Osteochondral Lesion of the Talus With a Subchondral Cyst: A Case Report

**DOI:** 10.7759/cureus.116878

**Published:** 2026-09-24

**Authors:** Toshiaki Kiribayashi, Hayato Seki, Taiki Ueno, Takuma Yoshimoto, Kazuki Shimoonoda

**Affiliations:** 1 Faculty of Health Sciences, SBC Tokyo Medical University, Urayasu, JPN; 2 Department of Orthopedic Surgery, SBC Tokyo Medical University, Kamiaoki Clinic of Orthopaedic, Kawaguchi, JPN; 3 Center for Fundamental Education, Faculty of Health Sciences, SBC Tokyo Medical University, Urayasu, JPN

**Keywords:** anderson classification, conservative treatment, osteochondral lesions of the talus, scranton classification stage v, subchondral cyst

## Abstract

Osteochondral lesions of the talus (OLT) are increasingly recognized as resulting from trauma in a broad sense, including repetitive microtrauma, yet detailed reports of lesions accompanied by a subchondral bone cyst that are managed conservatively alone remain scarce, and no consensus exists regarding their optimal management. We report the case of a 59-year-old woman with chronic right ankle pain that had not improved despite prior follow-up and who had no clear history of trauma. Examination revealed no swelling, tenderness, or pain on supination. Plain radiography and MRI demonstrated a subchondral cystic lesion with bone marrow edema on the medial talar dome, corresponding to a Scranton stage V, Anderson stage IIA osteochondral lesion. Given tolerable symptoms, small cyst size, and absence of cartilage damage on MRI, conservative management with outpatient observation was planned; however, the patient did not return for scheduled follow-up. She re-presented after a prolonged interval, and follow-up radiography showed no significant progression of the lesion. Although follow-up imaging in this case showed no significant progression of the lesion, prior literature indicates that radiographic progression can occur even in patients with favorable clinical symptoms, underscoring the importance of sustained clinical and radiographic follow-up in patients with OLT managed conservatively, even when early symptoms and imaging appear reassuring.

## Introduction

Osteochondral lesions of the talus (OLT), historically referred to by various other disease names, are increasingly recognized as arising from so-called “trauma” in a broad sense, including repetitive microtrauma [1]. The medial-type lesion has been attributed to a compression injury occurring when supination and plantar flexion of the ankle combine with external rotation of the tibia [2], as well as to persistent, intermittent compression of the medial talar dome [3]. Although the mechanism of injury in patients without a clear history of trauma remains largely unclear, such lesions are reportedly more common in women, more often bilateral, and more frequently located medially [4]. Radiographic staging of OLT is most commonly performed using the Berndt and Harty classification, with the Scranton classification adding stage V for lesions accompanied by a subchondral bone cyst [5]; MRI-based staging is performed using the Anderson classification [6]. The presence of a subchondral cyst, as defined in Scranton stage V lesions, is clinically important because it reflects a more advanced stage of subchondral bone involvement that may be associated with a higher risk of treatment failure and progression to osteoarthritis, potentially influencing the choice between conservative and surgical management [5]. Reports describing in detail cases of Scranton stage V OLT treated conservatively alone are scarce, and no consensus has yet been reached regarding their optimal management. We herein report such a case, managed conservatively, together with a review of the relevant literature, to highlight the importance of long-term clinical and radiographic follow-up even when initial symptoms and imaging findings appear favorable.

## Case presentation

The patient was a 59-year-old woman whose chief complaint was right ankle pain. Her past medical history and medication history were unremarkable. She had previously experienced right ankle pain, but the pain did not improve despite follow-up, and she presented to our clinic in June 2024. At the initial examination, no obvious swelling or tenderness was noted, and no pain was induced by supination of the right ankle. There was no clear history of ankle sprain, and the patient reported no activities in daily life or at work that placed a load on the right ankle. A plain X-ray taken in 2015 had shown no obvious abnormality (Figure 1). At the initial consultation, an anteroposterior plain X-ray revealed a round radiolucent lesion with marginal sclerosis in the talar dome (Figure 2). MRI demonstrated a 6 × 6 × 6 mm subchondral cyst with bone marrow edema, predominantly on the medial aspect of the talar dome (Figure 3), consistent with a diagnosis of Scranton stage V [5] osteochondral lesion of the talus, Anderson stage IIA [6]. As the pain was tolerable, the cyst was small, and no obvious cartilage damage was observed on MRI, conservative treatment was indicated, and follow-up observation as an outpatient was considered; however, the patient did not return to our clinic after the diagnosis. The conservative management plan consisted of clinical and radiographic observation alone, without cast immobilization or bracing, given the mild symptoms at presentation. She presented again one year and four months later, and a follow-up anteroposterior plain X-ray revealed no significant expansion of the lesion (Figure 4).

**Figure 1:**
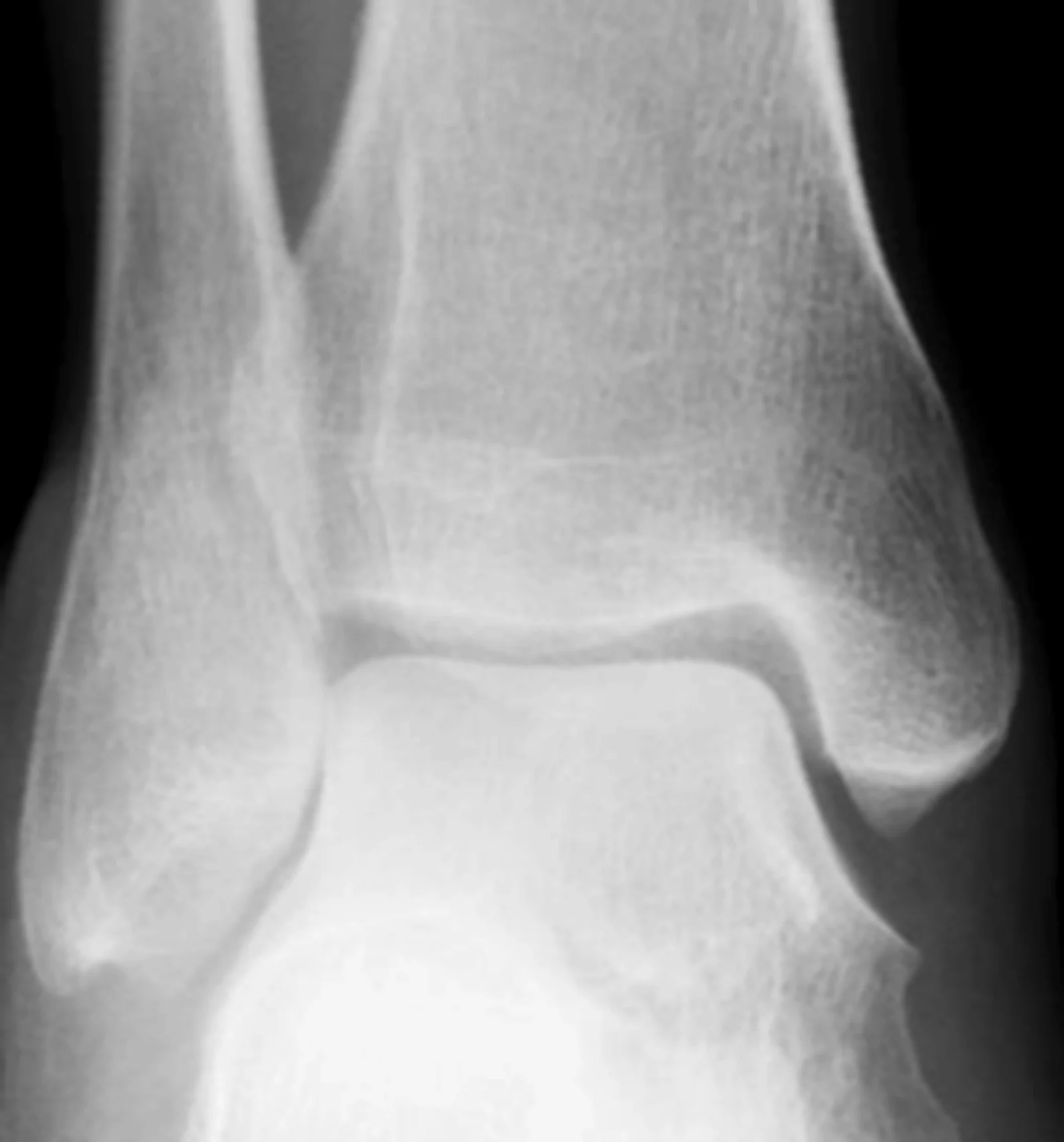
Anteroposterior plain X-ray taken in 2015 showing no obvious abnormal findings.

**Figure 2:**
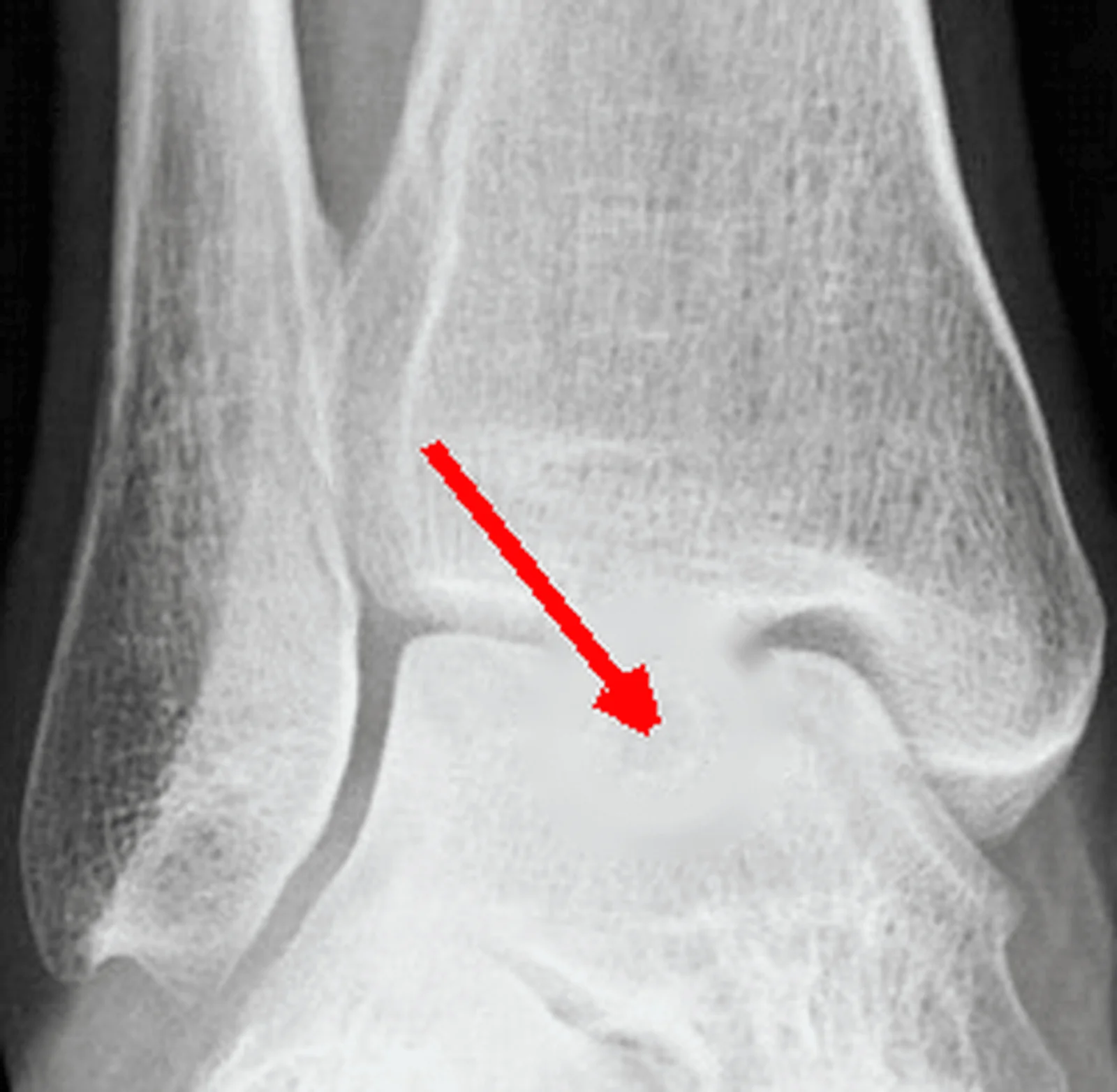
Anteroposterior plain radiograph obtained at the initial consultation showing a round radiolucent lesion with marginal sclerosis in the talar dome (arrow).

**Figure 3:**
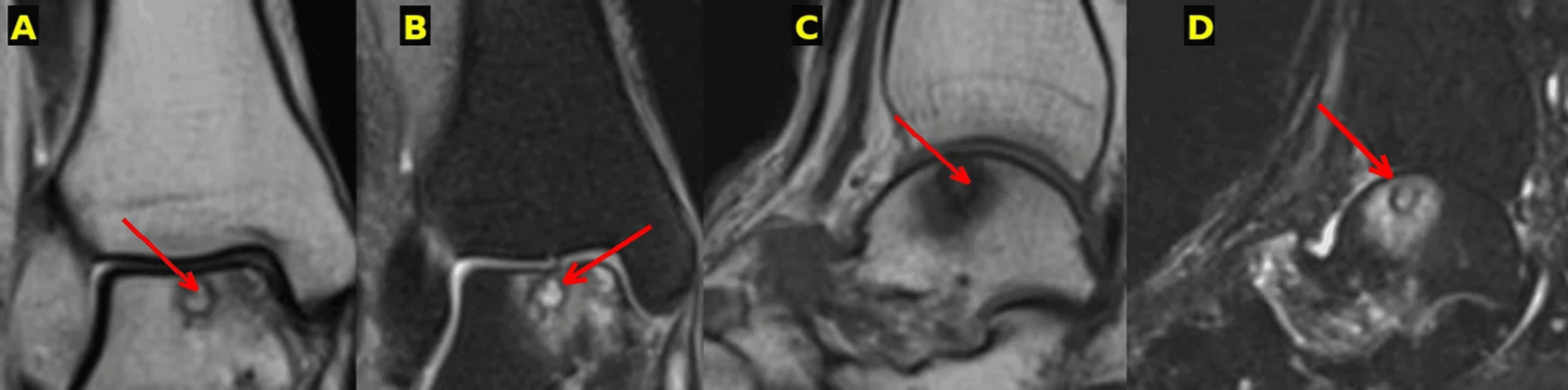
Right ankle MRI. (A) T1-weighted coronal image. (B) Fat-suppressed T2-weighted coronal image. (C) T1-weighted sagittal image. (D) Fat-suppressed T2-weighted sagittal image. Arrows indicate a 6 × 6 × 6 mm subchondral cyst with surrounding bone marrow edema on the medial aspect of the talar dome.

**Figure 4:**
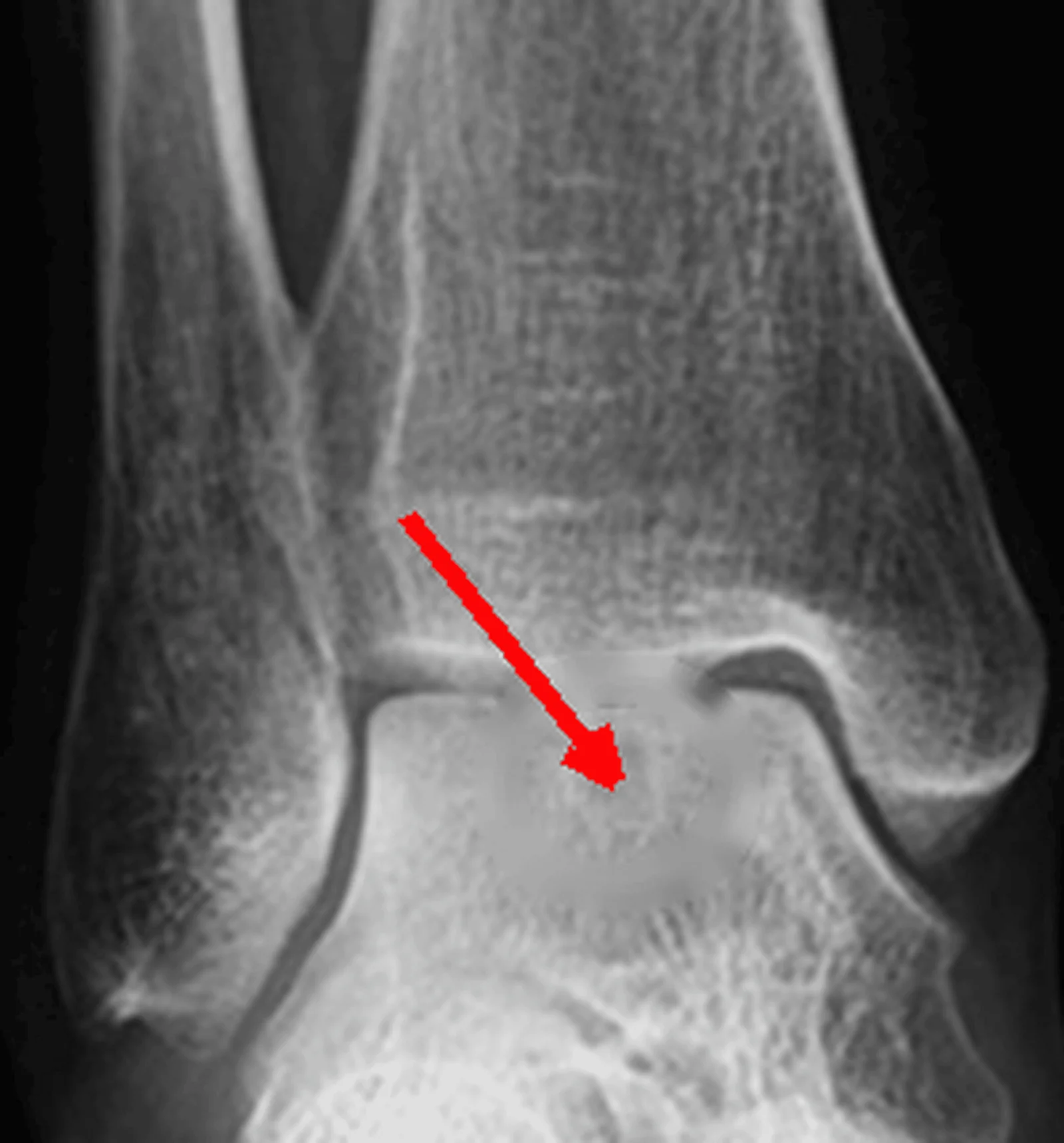
Anteroposterior plain radiograph obtained at follow-up showing no obvious expansion of the round radiolucent lesion (arrow).

## Discussion

In the present case, no clear precipitating trauma could be identified, which is consistent with previous reports describing a substantial proportion of OLT without an obvious traumatic history [4].

Conservative treatment options for OLT include cast immobilization and non-weight-bearing treatment using a patellar tendon-bearing (PTB) brace [7]. Singh et al. reported that in Scranton stage V cases treated with arthroscopic debridement, bone marrow stimulation (drilling), and injection of plasma rich in growth factors, the subchondral cyst disappeared, although progression of osteoarthritis was observed on imaging despite high patient-reported satisfaction (mean follow-up period: 29 months; mean patient age: 27.4 years) [8]. This discrepancy between clinical satisfaction and radiographic progression in the surgical literature may explain why our patient, despite tolerable symptoms, did not return for scheduled follow-up after the initial diagnosis. Although the follow-up radiograph in our patient fortunately showed no significant expansion of the lesion, the possibility of subclinical radiographic progression in patients who feel clinically well cannot be excluded over a longer time course, particularly given her age and the associated risk of osteoarthritis.

Limitations of this report include its single-case design and the interruption in the patient’s follow-up, which limited the ability to closely monitor the natural course of the lesion. In addition, detailed pain characteristics (e.g., duration, severity, and relationship to weight-bearing or activity) and a comprehensive functional examination, including range of motion, ligamentous stability, and gait assessment, were not systematically documented at the initial consultation, which may limit the reproducibility of the clinical presentation described. Nonetheless, this case underscores the importance of educating patients on the need for continued follow-up regardless of symptomatic improvement and highlights an area in which further prospective studies are needed to establish optimal management strategies for Scranton stage V lesions treated conservatively.

## Conclusions

We report a case of an osteochondral lesion of the talus managed conservatively, in which follow-up imaging performed after a prolonged interval showed no significant progression of the lesion despite an interruption in scheduled follow-up. Although this patient’s lesion remained radiographically stable despite an interruption in scheduled follow-up, favorable clinical symptoms do not always correspond to radiographic stability, as reported in the surgical literature. This case underscores the importance of maintaining regular clinical and radiographic follow-up in patients with OLT managed conservatively, even when initial findings appear reassuring.
